# CJP Special Edition—Proceedings of the Michael G. Degroote Institute for Pain Research and Care Annual Symposium—Director’s introduction

**DOI:** 10.1080/24740527.2020.1854043

**Published:** 2020-12-30

**Authors:** Norm Buckley

**Affiliations:** Michael G. DeGroote Institute for Pain Research and Care

We had the pleasure of welcoming many pain research leaders and specialists to our 2019 Annual Symposium, sponsored and held by the Michael G. DeGroote Institute for Pain Research and Care with the generous funding from Mr. DeGroote, whose support has permitted a broad range of activities that have already had great impact on pain care in Canada. We are very appreciative of the support from Joel Katz, Rebecca Lewinson, the editorial team, and peer reviewers in allowing us once again to present this special edition highlighting the discussions at the 2019 Annual Symposium of the Michael G. DeGroote Institute for Pain Research and Care. I would also like to thank our own Dr. Victoria Borg Debono for her work overseeing the submission of the articles you see here. The papers in this edition are from presentations at the symposium held in November 2019, an excellent in-person gathering and discussion around a wide range of topics relevant to persistent postsurgical pain, the construct we have used to guide our research.

Unfortunately, because of the COVID-19 pandemic, we have deferred the 2020 Annual Symposium in the hope that we will once again be able to hold an in-person meeting in 2021. Our small meeting has a very high value in terms of its collegiality and interpersonal interactions and thus far we have not seen virtual meetings that offer the same degree of intimacy, although we have certainly seen great examples of ingenuity and logistical expertise in presenting insightful meetings: The Chronic Pain Network is presenting a fascinating series of webinars, as are the Canadian Pain Society and the newly created Chronic Pain Center of Excellence for Canadian Veterans. The Pain Society of Alberta held a tour de force 3-day scientific meeting with over 4000 registrants, and there have been very productive virtual working meetings of the Canadian Pain Task Force and its external advisory panel, which has just released its second report on the state of pain care, awareness, research, and education in Canada. Great days ahead—possibly the most exciting time in the past 30 years for pain in Canada.

In this special issue, we offer a sampling of the topics that were presented during our 1-day symposium in November 2019. You will see keynotes from invited speakers and our scientific advisory board and reports of work supported by the institute. Dr. Jennifer Rabbits from the Seattle Children’s Hospital presented on pain trajectories following major musculoskeletal surgery in adolescents and stimulated animated discussion among our attendees. Her presentation has been distilled into the article presented here, and we look forward to the fully realized trial to come. Scientific Advisory Board Member Dr. Mark Lema from the State University of New York at Buffalo and the Roswell Park Cancer Institute updated us about progress in understanding mechanisms of chronic pain, with an eye to identifying new areas for therapeutic research and strategies. He and his colleague Dr. Cohen have distilled that talk into the article you will find in this issue.

One of the very challenging clinical problems in pain care is dealing with the effects of spinal stenosis. Dr. Luciana Macedo of the McMaster School of Rehabilitation Science has received seed grant funding to analyze historical data about spinal stenosis from the Canadian Spine Outcomes Research Network database and reports on this protocol here. Dr. Cheryl Chow, a postdoctoral researcher in the McMaster Department of Psychology, has a research program examining pediatric perioperative anxiety. The review she presents here is an extension of that body of work, specifically examining the effects of anxiety on persistent postsurgical pain in pediatrics. This review highlights several areas for future work to address the significant gaps in the literature. Finally, doctoral student Chad Brown, under the supervision of Dr. Karun Singh of the Stem Cell Research Institute, reports on a novel exploration of work connecting the genetics of autism and pain in developing a neuronal cell model upon which to test novel therapeutics.

All things considered, this is a wide-ranging collection of interesting papers, and we hope that they will stimulate further discussion, interest, and research. We look forward to building on the work represented in these papers. Even more so, we look forward to our next opportunity to convene the Michael G. DeGroote Institute for Pain Research and Care Annual Symposium and discuss the problem of persistent postsurgical pain and how we can advance our understanding and treatments as we struggle to more effectively deal with chronic pain in general. Thank you for your attention and interest.

